# Reptile research shows new avenues and old challenges for extinction risk modelling

**DOI:** 10.1371/journal.pbio.3001719

**Published:** 2022-07-11

**Authors:** Moreno Di Marco

**Affiliations:** Department of Biology and Biotechnologies, Sapienza University of Rome, Rome, Italy

## Abstract

This Primer explores the implications of a new PLOS Biology study that presents an innovative method for estimating extinction risk in reptile species worldwide; this method represents a promising avenue to support Red List assessment, alongside some well-known challenges.

## Teaser

In a new *PLOS Biology* paper, de Oliveira Caetano and colleagues presented an innovative method to estimate extinction risk in reptile species worldwide. The method shows a promising avenue to support Red List assessment, alongside some well-known challenges.

Human-induced rates of species extinction largely surpass the background rates registered from the fossil record [1], and global monitoring of extinction risk is essential to track progresses towards sustainable development. The Red List of the International Union for the Conservation of Nature (IUCN; hereafter “Red List”) is the global authority that manages data on species extinction risk, now including over 140,000 assessed species. Yet, while the taxonomic coverage of the Red List has rapidly grown, a parallel increase in resources for update (i.e., periodic reassessment) has not followed [2]. Limited reassessment efforts means that the Red List is constantly facing a risk of becoming outdated, with many species (ca. 20% at the time of writing) having an assessment older than 10 years and possibly undergoing undetected decline. Under rapidly accelerating human pressure, there is a clear need to make the global monitoring of extinction risk more effective.

Many works have proposed approaches that might support extinction risk monitoring [3] using automated estimates of Red List parameters, e.g., population decline inferred from satellite-borne estimates of deforestation rates [4], or directly modelling Red List categories (or aggregation of categories) from environmental and life history variables [5]. Yet, very few of these approaches have fed into the Red List process, generating a research-implementation gap [3]. For example, most extinction risk modelling exercise do not reflect the process of Red List assessment (including its required parameters and guidelines), which makes it difficult to incorporate modelling outputs in the Red List. At the same time, there is often an implementation barrier even for potentially relevant methods, due to limited technical capacity by (and limited training offered to) assessors. However, recent research on reptiles shows a promising avenue to advance this debate.

In a new *PLOS Biology* paper, de Oliveira Caetano and colleagues [6] presented an innovative machine learning analysis to estimate the extinction risk of 4,369 reptile species that were unassessed or data deficient in the Red List. Meanwhile, in a recent *Nature* paper, Cox and colleagues [7] presented the results of the Global Reptile assessment, including extinction risk categories for ca. 85% of the 10,196 reptile species in the Red List (the rest being data deficient). Reptiles are a diverse group which represent a perfect example of the “update or outdate” conundrum in the Red List, as their assessment required nearly 50 workshops and 15 years to complete [7]. At the same time, enough data on reptile distribution and life history are now available [8] to attempt large-scale extinction risk modelling for the group, indicating that it might be time to “bridge” the research-implementation gap [3].

**Fig 1:**
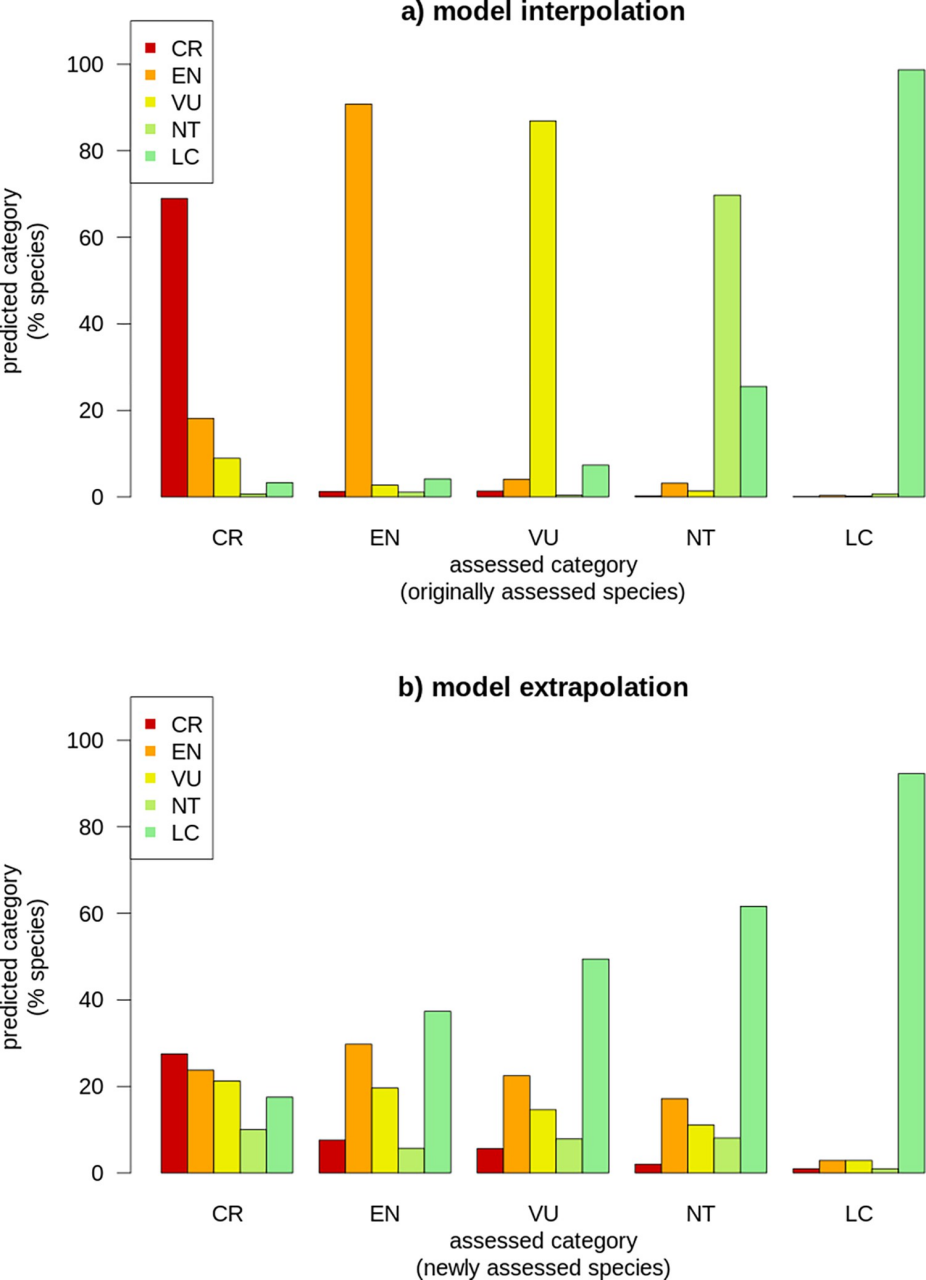
Comparison between the performance of the automated assessment model presented in [6] during interpolation and extrapolation. The bar plots report the contingency distribution between predicted Red List categories (y-axis, prediction) and assessed categories (x-axis, observation). Plot **(a)** reports the contingency between assessed versus predicted categories for 6,520 species used to train the automated assessment model in [6]. Plot **(b)** reports the contingency between assessed versus predicted categories for 1,463 species that were considered unassessed and not used for model training in [6] and were only assigned a Red List category in 2021 [9]. For this latter comparison, I only selected species having precise taxonomic correspondence with the latest release of the IUCN Red List database and being assigned a category of risk (see S1 Table), as follows: CR, critically endangered; EN, endangered; LC, least concern; NT, near threatened; VU, vulnerable.

The model presented in [6] was 84% accurate in predicting Red List categories during cross-validation and found unassessed species to face higher risk compared to assessed species (27% versus 21% species threatened with extinction). The model’s performance was higher compared to previous similar exercises, albeit prediction accuracy for certain categories (e.g., near threatened) was substantially lower than others (e.g., least concern). The recent completion of nearly all reptile assessments in the Red List [9] allows to compare the model’s performance measured on the training set of originally assessed species (i.e., “model interpolation”) versus the performance measured on newly assessed species not used for model training (i.e., “model extrapolation”) (Fig 1).

The automated assessment model in [6] showed high accuracy both in the interpolation and extrapolation of least concern species: 92% of the species newly assessed as least concern were correctly predicted by the model. This reflects the ability of automated methods to separate least concern species from the rest, which is a promising implementation for facilitating periodic reassessments [10]. However, the model’s ability to extrapolate near threatened and threatened categories was substantially lower than the ability to interpolate those categories. Less than 30% of the newly assessed species in each of these categories were correctly predicted by the model: In most cases, these species were predicted as least concern.

The mismatch between predicted versus assessed categories during model extrapolation can have multiple causes. For 18% newly assessed species, the model predicted a lower category of risk than what Red List assessors have then assigned. This might happen because assessors have access to information on threats that are not explicitly accounted for in the model (harvesting, pathogens, invasive species, etc.). Instead, for 10% of species, the model predicted a higher category of risk than that assigned by Red List assessors. This might be related to the compound mechanistic nature of Red List criteria, which require a combination of parameters that models are typically unable to account for (e.g., restricted distribution AND severe fragmentation AND continuing decline). Importantly, however, the 2 works are based on different sources of species’ distribution maps, which can lead to a discrepancy in the measure of environmental and spatial variables (e.g., extent of occurrence) for the same species. If the distribution maps of newly assessed species differ substantially between the GARD dataset [8] and the Red List dataset [9], the mismatch in category prediction can be simply an outcome of different underlying data. This calls for a better homogenisation of spatial data used for extinction risk modelling and assessment purposes. Of course, there is also the possibility that some of the new assessments are incorrect, as Red List assessors did not have sufficient information to determine a species’ status while the model was able to use ancillary information. In this case, an indication of mismatch between predicted versus assessed category can be used to inform future reassessments [3].

Regardless of prediction performance, both recent works [6,7] highlight the difficulty to properly account for the effect of climate change. Cox and colleagues acknowledged the limited consideration of climate vulnerability in reptile Red List assessments [7], as the proportion of threatened species at risk from climate change (11%) was much lower than that of birds (30%). This likely indicates lower knowledge rather than lower vulnerability, considering that reptiles are ectotherms with limited climatic tolerance and dispersal ability [11]. Possibly because of this knowledge gap, climatic variables had limited predictive importance in the automated assessment model in [6]. As climate change accelerates, it is paramount that climate risk for groups such as reptiles and amphibians is consistently and customarily assessed in the Red List [12].

The recent publication of an innovative extinction risk model, alongside the complete Red List assessment of reptile species, shows promising avenues but also some well-known challenges for technological applications in the Red List. Automated assessment models can help Red List assessors by (i) quickly identifying species that are least concern and not in need of immediate conservation attention; (ii) pinpointing species that might be in need of reassessment (i.e., those with a mismatch between predicted versus assessed category); and (iii) investigate any significant bias in the assessment process (e.g., associated with differential application of the Red List guidelines by assessors). However, for these methods to be effective, it is important that model outputs are shared with assessors and any feedback is iteratively used to improve model’s structure, interpretation, and validation.

## Supporting information

S1 Table List of reptile species considered unassessed (and not used for model training) in the work of de Caetano Oliveira and colleagues and subsequently assigned a Red List category in 2021. The list only includes species having precise taxonomic correspondence with the latest release of the IUCN Red List database and being assigned a category of risk.
(XLSX)
